# An Evaluation of the Quality of IMCI Assessments among IMCI Trained Health Workers in South Africa

**DOI:** 10.1371/journal.pone.0005937

**Published:** 2009-06-17

**Authors:** Christiane Horwood, Kerry Vermaak, Nigel Rollins, Lyn Haskins, Phumla Nkosi, Shamim Qazi

**Affiliations:** 1 Centre for Rural Health, University of KwaZulu-Natal, Durban, South Africa; 2 Department of Paediatrics and Child Health, University of KwaZulu-Natal, Durban, South Africa; 3 Department of Child and Adolescent Health and Development, World Health Organisation, Switzerland, Geneva; JARING, Malaysia

## Abstract

**Background:**

Integrated Management of Childhood Illness (IMCI) is a strategy to reduce mortality and morbidity in children under 5 years by improving case management of common and serious illnesses at primary health care level, and was adopted in South Africa in 1997. We report an evaluation of IMCI implementation in two provinces of South Africa.

**Methodology/Principal Findings:**

Seventy-seven IMCI trained health workers were randomly selected and observed in 74 health facilities; 1357 consultations were observed between May 2006 and January 2007. Each health worker was observed for up to 20 consultations with sick children presenting consecutively to the facility, each child was then reassessed by an IMCI expert to determine the correct findings. Observed health workers had been trained in IMCI for an average of 32.2 months, and were observed for a mean of 17.7 consultations; 50/77(65%) HW's had received a follow up visit after training. In most cases health workers used IMCI to assess presenting symptoms but did not implement IMCI comprehensively. All but one health worker referred to IMCI guidelines during the period of observation. 9(12%) observed health workers checked general danger signs in every child, and 14(18%) assessed all the main symptoms in every child. 51/109(46.8%) children with severe classifications were correctly identified. Nutritional status was not classified in 567/1357(47.5%) children.

**Conclusion/Significance:**

Health workers are implementing IMCI, but assessments were frequently incomplete, and children requiring urgent referral were missed. If coverage of key child survival interventions is to be improved, interventions are required to ensure competency in identifying specific signs and to encourage comprehensive assessments of children by IMCI practitioners. The role of supervision in maintaining health worker skills needs further investigation.

## Introduction

In developing countries 9.7 million children under five years of age die every year [1], most deaths are from preventable and easily treatable diseases [2], and in a small number of developing countries [3]. It is estimated that over 60% of global child deaths could be prevented by available and affordable interventions [4], and their effective delivery is critical for achieving the Millennium Development Goal for child survival [5]. Integrated Management of Childhood Illness (IMCI) is a child survival strategy developed by the World Health Organisation (WHO) and United Nations Children's Fund (UNICEF) [6]. IMCI aims to improve coverage of essential child health interventions by improving case management skills of first level health workers, strengthening the health system for effective management of sick children, and promoting good community child care practices [7]. South Africa adopted IMCI as the standard of care for children in 1997, and is one of 43 African countries to do so [8].

IMCI case management training equips health workers with skills to manage children for a combination of illnesses, identify those requiring urgent referral, administer appropriate treatments, and provide relevant information to child carers. WHO recommends that newly trained IMCI practitioners receive follow-up visits from IMCI supervisors, starting 4–6 weeks after training, to assist them in transferring their newly acquired skills to the workplace [9]. IMCI implementation has been shown to improve the quality of management of sick children [10], [11], [12], and IMCI trained health workers communicate better with care-givers [13].

However, previous reviews have not described healthworkers' assessments of children in detail, and have used the observed child as the unit of analysis. In this article we report the results of an evaluation of the performance of IMCI trained health workers, conducted in two provinces in South Africa. We undertook a large number of observations overall, and for each health worker, so we are able to use the health worker as the unit of analysis and describe in detail how health workers assess and classify sick children. This provides a comprehensive picture of IMCI implementation in routine clinical practice, from which we are able to identify gaps in implementation, and suggest solutions.

## Methods

### Study site and population

IMCI guidelines in South Africa were adapted to include a component for management of HIV infected children, and evaluation of this component was a primary objective of this study. IMCI trained health workers (HW's) working in first level health facilities in Limpopo and Kwazulu-Natal (KZN) provinces, South Africa, were randomly selected for inclusion in the study. Health workers without IMCI training were excluded. IMCI implementation began in 1998, and at the time of our study 1325 health workers had been trained in Limpopo Province and 1300 in KZN, comprising 47% and 32% of health workers seeing sick children in PHC clinics respectively.

### Training and Data Collection

Two IMCI experts visited facilities to collect data in each province. They were trained in study methods for two weeks by the investigators (CH, KV); data collection tools and methodology were piloted in two health facilities. All IMCI experts had previously attended the 11 day IMCI training course and the IMCI facilitators' course, and were experienced IMCI course directors.

The consultation by the health worker was observed by one IMCI expert who recorded the activities and findings without intervening. Activities recorded included whether the health worker referred to the IMCI chart booklet during the consultation, as would be expected if IMCI was being implemented correctly. During an IMCI consultation, health workers assess first for general danger signs, they then assess the four main symptoms (cough or difficult breathing, diarrhoea, fever, ear pain) and nutritional status. A classification is then made for each main symptom present, according to the signs identified during the assessment of the child. Thereafter, the second IMCI expert assessed the same child independently, and these findings were considered to be the gold standard for analysis of health worker performance. If the management of the child was incorrect, this was changed by the second IMCI expert as appropriate. Each health worker was observed for 20 consultations with sick children aged 2–59 months presenting consecutively to the health facility, or for 3 days if 20 observations had not been completed in that time.

The IMCI experts used standardized data collection instruments to record data about health workers' previous training and supervision; assessments by the health worker and the IMCI expert; and resources available at the clinic to support IMCI implementation. To monitor quality during data collection, the principal investigator visited the teams at least monthly, and all completed forms were checked for quality and completeness.

### Consent and ethical approval

Written informed consent was obtained from carers of children for observation of the consultation with the health worker, and for the second assessment of the child by the IMCI expert. Health workers and observed children were allocated codes and no identifying information was recorded.

The study was conducted in partnership with the South African Department of Health (DOH), and all first level health workers in the two provinces were informed by the DOH that a survey of child health practices was to be undertaken. Participants were not told ahead of time that they had been selected, or that IMCI in particular was being evaluated, and because they were employed by the DOH they were required to participate.

Ethical approval was obtained from the Biomedical Research Ethics Committees of the University of KwaZulu-Natal Medical School, Durban, and WHO, Geneva.

### Sample Size

A major objective of this study was to assess implementation of the IMCI HIV component. The sample size calculation was based on the assumption that 80% (+/−10%) of health workers would correctly classify for HIV in all 20 cases they assessed, compared to the IMCI expert. The sample was calculated as 62 health workers. IMCI trained health workers were randomly selected from a list of all IMCI trained health workers in each province using computer generated random numbers [14]. Sampling was stratified by province with equal numbers of health workers selected from each province.

However, an interim analysis found that only 26% (+/−10%) of health workers had correctly classified all children, the sample was therefore recalculated and increased to 77 health workers. The results of the HIV implementation assessment are reported elsewhere.

### Data management and analysis

Pre-coded data were double entered, cleaned and validated using Epi-info (version 6.04). Analysis was conducted using SPSS (version 13.0) and Stata (version 9). The proportion of health workers who referred to the chart booklet and how frequently, was used as an indicator of whether observed health workers were implementing IMCI. To determine the performance of observed health workers, their assessments were compared to those made by the IMCI experts, which were considered to be ‘correct’ for purposes of analysis. To assess the performance of each health worker during the period of analysis, the proportion of observed children correctly assessed for each main symptom was calculated for each health worker. Using the child as the unit of analysis, we then calculated the proportion of children with each main symptom who were assessed correctly, assessed incorrectly or not assessed at all, by observed health workers. We then calculated the proportion of children with each IMCI classification that were correctly classified by observed health workers, using the child as the unit of analysis. 95% confidence intervals were calculated for all performance indicators.

## Results

The consultations of 77 IMCI trained health workers working in 74 primary health care clinics in KZN and Limpopo provinces were observed between May 2006 and January 2007. Each health worker was observed for a mean of 2.7 days and 17.7 consultations.

### Training of observed health workers

All observed health workers were registered nurses with a minimum of 3 years nursing training, and had attended an 11day IMCI training course, but most had no other special training in child health. The time since being trained in IMCI was an average of 32.2 months. Most health workers had received at least one follow up visit following IMCI training (table 1).

**Table 1:** Training of observed health workers.

Months since training in IMCI (n = 77)	Number (%)
1–11	12 (16)
12–23	10 (13)
24–35	22 (29)
36–47	16 (21)
48 or more	17 (22)
**Additional training in child health**	
None	55 (71)
IMCI facilitator	1 (1)
IMCI supervisor	1 (1)
Primary health care diploma^a^	7 (9)
Expanded programme of immunisation	10 (13)
Anti-retroviral treatment for children	1 (1)
Tuberculosis treatment for children	1 (1)
**Number of IMCI follow-up visits**	
0	27 (35)
1	34 (44)
2	13 (17)
3	3 (4)

a One year course includes paediatric module.

The average number of nurses on the staff establishment at clinics where we undertook our observations was 6, and on average 74% of these had been trained in IMCI. In 50/74 (67%) clinics visited, more than 60% of nurses were IMCI trained.

### Performance of observed health workers during the observation period

31/77(40%) health workers referred to the IMCI chart booklet during every observed consultation, 35(45%) did so during some observed consultations, and only one health worker never referred to the chart booklet during the period of observation.

During the period of observation, 9 (12%) health workers asked about three general danger signs (unable to drink or breastfeed, vomiting everything, and convulsions with this illness) in every child, and 14 (18%) asked about all four main symptoms in every child. 7/9 (78%) health workers who checked the danger signs in every child also checked all main symptoms in every child. Only 17 (22%) health workers plotted the weight of all children. Depending on the presenting complaints of children presenting to the facility, each observed health worker assessed children with different symptoms and signs. Table 2 shows the performance of each health worker in classifying the children assessed during the observation period.

**Table 2:** Proportion of children with each main symptom assessed correctly by health workers' during the observation period.

Main symptom n = 77^a^	No of observed health workers^b^	No of health workers who assessed >80% children correctly (%) (95% CI)	No of health workers who assessed 60–80% children correctly (%) (95% CI)	No of health workers who assessed <60% children correctly (%) (95% CI)
Cough or difficult breathing	77	9 (12) (6–21)	35 (46) (35–57)	33 (43) (32–54)
Diarrhoea/dehydration	72^c^	20 (28) (18–40)	21 (29) (20–41)	31 (43) (32–55)
Fever	76^d^	14 (18) (11–29)	9 (12) (6–22)	53 (70) (58–79)
Ear problem	64^e^	10(16) (9–27)	5 (8) (3–18)	49 (77) (64–86)
Nutritional assessment (all children)	77	4 (5) (2–13)	10 (13) (7–23)	63 (82) (71–89)
Any severe classification	54^f^	14 (26) (8–24)	3 (6) (5–20)	36 (68) (54–80)

a Unit of analysis is the health worker.

b Observed health workers saw a different number of children with each of the main symptoms.

c Excludes 5 health workers who did not see any child with diarrhoea.

d Excludes 1 health worker who did not see any child with fever.

e Excludes 13 health workers who did not see any child with an ear problem.

f Excludes 24 health workers who did not see a child with any severe classification.

No association was found between health worker performance and whether the health worker had received a follow up visit by a supervisor, or the time since being trained in IMCI (data not shown). However the number of health workers in the sample was insufficient to exclude such an association.

### Classification of observed children by health workers

During the 1357 observed consultations, health workers asked about three general danger signs in 795(58.6% CI: 49.8%–66.9%), and the four main symptoms in 815(60.1% CI: 51.0%–68.5%) children. Health workers did not ask about cough in 123(9.1% CI: 6.3%–12.9%) children, diarrhoea in 297(21.9% CI: 15.7%–29.6%) children, fever in 310(22.8% CI: 17.1%–29.8%) children, and ear problems in 409 (30.1% CI: 23.0%–38.3%) children. The performance of health workers in classifying observed children for each main symptom is shown in table 3.

**Table 3:** Health worker (HW) performance in classifying children with each of the main symptoms.

N = 1357^a^	Cough (%) n = 1076^b^ (95% CI)	Dehydration (%) n = 311^b^ (95% CI)	Fever (%) n = 789^b^ (95% CI)	Ear problem (%) n = 151^b^ (95% CI)	Malnutrition (%) n = 1212^c^ (95% CI)
Symptom not reported to HW^d^	38 (3.5) (2.4–5.1)	25 (8.0) (5.2–12.2)	125 (15.8) (12.5–19.9)	31 (20.5) (14.9–27.6)	n/a
HW did not ask about symptom	30 (2.8) (1.9–4.1)	14 (4.5) (2.4–8.2)	121 (15.3) (11.3–20.5)	21 (13.9) (8.7–21.5)	130 (10.7) (7.2–15.6)
HW asked about symptom and/or assessed child but did not classify	106 (9.8) (7.1–13.5)	46 (14.8) (10.9–19.7)	212 (26.9) (20.9–33.8)	22 (14.6) (9.6–21.5)	437 (36.1) (29.8–42.8)
Incorrectly classified by HW	245 (22.8) (20.3–25.4)	29 (9.3) (6.3–13.6)	1 (0.1) (0.0–0.9)	23 (15.2) (10.2–22.2)	177 (14.6) (11.2–18.6)
Correctly classified by HW	645 (59.9) (56.0–63.8)	195 (62.7) (56.6–68.4)	312 (39.5) (31.9–47.8)	51 (33.8) (26.0–42.5)	446 (36.8) (30.7–43.4)

a Unit of analysis is the child.

b A total of 1357 consultations were observed but different numbers of children presented with each main symptom.

c Excluded 145 children where there was no chart available from the mother documenting weight for age.

d Carer reported symptom to IMCI expert but not to the health worker when asked.

Of 112 children assessed as having a severe classification or a danger sign by the IMCI expert, 52 (46.4% CI: 35.5%–57.7%) were also given a severe classification by the health worker. Health workers' performance in identifying each IMCI classification is shown in table 4.

**Table 4:** Proportion of classifications correctly identified by health workers.

Correct Classification (from IMCI expert)	Number of children with classification	Number correctly identified by health worker (%)	95% Confidence intervals
**Cough or difficult breathing (n = 1076^a^)**			
Severe pneumonia or very severe disease	69	33(47.8)	34.6–61.4
Pneumonia	360	146(40.6)	34.1–47.4
Cough or cold	645	466(72.2)	67.4–76.7
**Total**	1074^b^	645 (60.0)	56.0–63.8
**Dehydration (n = 311^a^)**			
Severe dehydration	3	1(33.3)	4.1–85.5
Some dehydration	37	14 (37.8)	22.0–56.8
No visible dehydration	270	180 (66.7)	59.7–73.0
**Total**	310^c^	195 (62.9)	56.6–68.4
**Fever (n = 789^a^)**			
Suspected meningitis	11	4 (36.4)	12.5–69.5
Fever other cause	776	308 (39.7)	31.8–48.2
**Total**	787^b^	312 (39.6)	31.9–47.8
**Malnutrition (n = 1357 a)**			
Severe malnutrition	18	5(27.8)	12.2–51.5
Not growing well	478	145(30.3)	24.1–37.4
Growing well	715	296 (41.4)	32.8–50.5
**Total**	1211^d^	446 (36.8)	30.7–43.4

a denominator different for each main symptom according to the number of observed children with that symptom.

b 2 missing,

c 1 missing,

d 1missing, 145 could not be classified because there was no chart documenting weight for age.

Health workers either did not assess, or did not classify, for malnutrition in 567/1357(41.8% CI: 34.2%–49.8%) children (table 3), but the weight was plotted correctly on the growth chart in 1060/1357(78.1% CI: 72.9%–82.5%) children. The findings were explained to the mother in only 624(58.9% CI: 52.7%–64.8%) cases.

### Feeding assessments

IMCI requires that all children under 2 years, and any who are low weight for age, should have a feeding assessment. Of 944 children required a feeding assessment according to these criteria, this was completed in 630 (66.7% CI: 60.1%–72.8%) children.

## Discussion

Our findings show that IMCI is being widely implemented in clinics in South Africa several years into the expansion phase. Most clinics visited had good coverage with IMCI trained health workers, and despite the average time since training being almost three years, all but one health worker used the IMCI guidelines during observed consultations. However the IMCI assessment was not applied consistently and comprehensively, and activities not related directly to the presenting complaint were frequently omitted.

More observations were done in this study, both in total and of each health worker, than previous IMCI evaluations, allowing us to describe health worker performance in more detail. Health workers' performed best in assessing cough and dehydration, but even with these symptoms, only a small proportion of health workers assessed more than 80% of children correctly. The most common reason for health workers' not classifying correctly was failure to ask about the symptom or to make a classification at all, rather than making an incorrect classification. Few health workers consistently asked about all main symptoms, particularly later in IMCI assessment, indicating that incomplete assessments rather than simply lack of skills often leads to poor IMCI implementation.

Health workers' performance in identifying different classifications shows that health workers frequently fail to identify children with moderate or severe classifications, and perform best at identifying common, mild illnesses where no specific treatment is required. Less than half of severely ill children who required urgent referral to hospital were identified by IMCI trained health workers. Correct assessment of moderate or severe classifications depends on health workers' ability to identify specific signs, whereas mild classifications are usually based on the absence of these signs. For example, when assessing a child with a cough, identification of fast breathing or chest indrawing leads to a classification of pneumonia or severe pneumonia, whereas failure to identify these signs would lead to the mild classification of no pneumonia: cough or cold. It may be a lack of skills in identifying those specific signs required to make severe classifications that leads to poor performance, so those children most at-risk do not receive appropriate treatment.

Nutritional assessments were also poorly implemented; many children were not assessed for nutrition, most children with malnutrition were not identified, and feeding advice was frequently not given where indicated. Interventions and advice about nutrition, particularly promotion of breastfeeding and counselling about complementary feeding, have been shown to substantially improve child mortality [15]. Thus, failure to implement this aspect of IMCI will have a major impact on the potential for the IMCI strategy to improve child survival. A review of training materials and methods related to nutrition and identification of children with severe illnesses, could improve performance in these important areas of practise.

IMCI has been shown to improve care of children at first level [10], [11], [12], but poor adherence to IMCI guidelines has been repeatedly described [12], [16], [17], [18]. If IMCI implementation is to achieve sufficient coverage to make a difference to child mortality, it is critical that strategies are developed to achieve and maintain high quality health worker performance. Our results suggest that strategies to encourage health workers to apply the IMCI assessment comprehensively, including the nutritional assessment, would lead to an improvement in health worker performance.

Our results also highlight the importance of health workers' achieving competency at identifying signs of severe disease during IMCI training. Previous evaluations have shown that health worker performance is adversely affected when the amount of clinical practice included in IMCI training is reduced [16], as may occur when training is decentralised. So interventions to improve health worker performance should include ways of ensuring that competency in identifying the severe signs used in the IMCI assessment is achieved and maintained. A formal assessment could be introduced for IMCI practitioners on completing the training, and regular updates for IMCI practitioners could ensure that these skills are maintained, as well as providing support for practitioners in the workplace. Other methods of improving implementation like awarding clinics ‘IMCI excellent’ accreditation could be used to motivate practitioners.

The strengths of this study are that we observed large numbers of health workers and for more consultations than previous evaluations of IMCI implementation, so that analysis could be done at the health worker level. All our IMCI experts were experienced IMCI facilitators, and able to provide a reliable gold standard. The influence of the observer's presence on health worker performance was minimised by the large number of observations conducted over several days, so subject bias was reduced by habituation. Limitations of the study include not evaluating health workers' ability to identify particular signs, or treatments given to children, and no measure was taken of inter-rater reliability. We were also unable to determine reasons for poor performance in sufficient detail, including any relationships that may have existed between health worker performance and IMCI supervision, or time since training.

Further research is required to investigate the factors leading to poor health worker performance, which is frequently ascribed just to a lack of knowledge and skills. Health workers often find it difficult to transfer new skills to the work place, and to maintain these skills, especially as IMCI consultations take longer [19]. Implementing and sustaining IMCI follow up after training has been shown to be difficult in several previous evaluations of IMCI [11], [12], [16], [20]. However, supervision has been shown to improve performance [11] and may also improve motivation and job satisfaction. The role of IMCI supervision in IMCI implementation and different models for provision of supervision should be investigated further.

In conclusion, IMCI can improve quality of care for sick children, and is being implemented in those countries where most child deaths occur. In our setting almost all IMCI trained health workers were using IMCI to assess children, but incomplete implementation means IMCI is failing to achieve maximum benefits for child survival. Improvements in training and supervision can go some way to addressing these problems, but further research is required to fully understand the determinants of health worker performance, both in the long and short term and strategies for maintaining IMCI skills over time should be evaluated. Effective solutions to the problem of scaling up IMCI, and other public health interventions, are needed to bridge the gaps between knowledge and practise, and to achieve universal coverage of critical interventions to improve child survival.
